# Editorial: Advances in buckwheat research

**DOI:** 10.3389/fpls.2023.1190090

**Published:** 2023-04-18

**Authors:** Artur Pinski, Meiliang Zhou, Alexander Betekhtin

**Affiliations:** ^1^Institute of Biology, Biotechnology and Environmental Protection, Faculty of Natural Sciences, University of Silesia in Katowice, Katowice, Poland; ^2^Institute of Crop Sciences, Chinese Academy of Agricultural Sciences, Beijing, China

**Keywords:** buckwheat, Fagopyrum (buckwheat) species, flavonoid biosynthesis, heterostylous self-incompatibility, CRISPR/Cas9

In recent years, buckwheat has gathered increasing attention as a promising functional food source, owing to its numerous human health benefits and absence of gluten. Furthermore, buckwheat species possess various properties that enhance habitats and associated biodiversity, making it an environmentally-friendly crop (Small, 2017). Additionally, buckwheat is considered an alternative to wheat for extreme drought conditions (Martínez-Goñi et al., 2023). Given these facts, it is imperative to conduct extensive molecular and field studies dedicated to fully realize the potential of this crop. The advancement of genetic studies and molecular breeding programs for buckwheat has been made possible through the gradual refinement of the genomes of Fagopyrum species, which was initiated with the release of the genome of *Fagopyrum esculentum* by Yasui et al. (2016), *Fagopyrum tataricum* cv. Pinku1 by Zhang et al. (2017), and *Fagopyrum cymosum* by He et al. (2022). The improved genome of *F. esculentum* cv. Dasha was later released by Penin et al. (2021) together with a high-resolution transcriptome atlas, including 46 organs and developmental stages. This transcription atlas constitutes a powerful tool for formulating and validating hypotheses regarding gene functionality in buckwheat. Utilizing these sequenced genomes, Zhang et al. (2021) reported a comprehensive database of Tartary buckwheat genomic variation based on whole-genome resequencing of 510 germplasms, while Zhao et al. (2023) described the seed metabolome during Tartary buckwheat domestication. Recently, high-quality genomes and chromosome-level genome assembly of *F. esculentum* cv. Pintian4 and *F. tataricum* cv. Pinku1 were made assessable to the research community (He et al., 2023).

The available genome sequence of *F. tataricum* was instrumental in the transcriptomic analysis of response to cadmium stress brought by Ye et al. This study’s findings reveal Tartary buckwheat’s tolerance to high cadmium concentrations (50 mg/L) and accumulation of the cadmium predominantly in the roots, with limited translocation to the upper portion of the plant. Together with a short life cycle, low nutritional requirements, and abundant biomass production, these features led authors to propose that Tartary buckwheat could be utilized in the phytoremediation of cadmium. Indeed, previous studies showed that Tartary buckwheat, due to its high tolerance, could be suitable for the phytoremediation of other heavy metals, e.g. lead, aluminum, and mercury (Pirzadah et al., 2018; Pirzadah et al., 2019; Wang et al., 2020). While Tartary buckwheat meets the criteria given for plants suitable for phytoremediation, more comprehensive field studies, including a combination of various contaminants, are needed to fully harness the potential of this species (Kafle et al., 2022). Besides the potential for phytoremediation, Fagopyrum plants contain phytoliths and phytolith-occluded organic carbon (PhytOC) that are relevant for carbon sequestration mechanisms, as described by Wang and Sheng. Thus, the cultivation of buckwheat has a phytolith carbon sink potential and can partake a significant role in carbon cycle regulation. The phytolith carbon sequestration of common and Tartary buckwheat were estimated to be equal to that of terrestrial shrub vegetation.

Most scientific attention on buckwheat species is focused on various medicinal and nutritional metabolites, predominantly flavonoids. To date, most of the studies focus on Tartary buckwheat due to its high content of flavonoids and their diversity, leading to well-described biosynthesis and regulatory mechanisms for this species (Zhang et al., 2017; Ding et al., 2021; Ding et al., 2022; He et al., 2023). The study by Huang et al. shifted the attention to rhizomes of Golden buckwheat (*Fagopyrum cymosum*) used in Traditional Chinese Medicine, describing its flavonoid biosynthesis pathway and regulatory network for the first time. Integrative transcriptome and metabolome analysis allowed the identification of SG5 R2R3-MYB transcription factor that plays the role of transcriptional activator in catechin biosynthesis. Presented here and other studies of flavonoid biosynthesis and its regulation in buckwheat species identified numerous genes that could be used to improve flavonoid production. In order to make full use of this knowledge, reliable protocols for the stable transformation of buckwheat species are needed. Moreover, successfully applying the CRISPR/Cas9 system could deepen our understanding of fundamental processes and the development of plant varieties with improved agronomical traits (Tomasiak et al., 2022). The detailed study by Sokoloff et al. on the inflorescence morphology of common buckwheat and its wild relatives established a formalized approach that can be applied in breeding buckwheat, its taxonomy, and evolutionary biology. The authors proposed that the Fagopyrum genus provides a suitable model for evolutionary developmental biology studies dedicated to inflorescence architecture, which could leverage recent advances in buckwheat genomes sequencing.

The articles gathered in Research Topic titled Advances in Buckwheat Research truly harness the outcomes of numerous studies of Fagopyrum genus bringing new and relevant results that will propel improvements of agronomical traits and a basic understanding of buckwheat biology. With growing research groups focusing on this magnificent genus, establishing a buckwheat community could facilitate further progress resulting in agriculture-relevant outcomes.

## Author contributions

All authors listed have made a substantial, direct, and intellectual contribution to the work and approved it for publication.
